# Mass spectrometry based proteomics profiling of human monocytes

**DOI:** 10.1007/s13238-016-0342-x

**Published:** 2016-11-22

**Authors:** Yong Zeng, Fei-Yan Deng, Wei Zhu, Lan Zhang, Hao He, Chao Xu, Qing Tian, Ji-Gang Zhang, Li-Shu Zhang, Hong-Gang Hu, Hong-Wen Deng

**Affiliations:** 1 College of Life Sciences and Bioengineering, Beijing Jiaotong University, Beijing, 100044 China; 2 Center of Bioinformatics and Genomics, Tulane University School of Public Health and Tropical Medicine, New Orleans, LA 70112 USA; 3 College of Life Sciences, Hunan Normal University, Changsha, 410081 China; 4 Laboratory of Proteins and Proteomics, Department of Epidemiology, Soochow University School of Public Health, Suzhou, 205123 China

**Keywords:** human monocytes, proteomics knowledgebase, gene ontology, gene-disease association, network analysis

## Abstract

Human monocyte is an important cell type which is involved in various complex human diseases. To better understand the biology of human monocytes and facilitate further studies, we developed the first comprehensive proteome knowledge base specifically for human monocytes by integrating both *in vivo* and *in vitro* datasets. The top 2000 expressed genes from *in vitro* datasets and 779 genes from *in vivo* experiments were integrated into this study. Altogether, a total of 2237 unique monocyte-expressed genes were cataloged. Biological functions of these monocyte-expressed genes were annotated and classified via Gene Ontology (GO) analysis. Furthermore, by extracting the overlapped genes from *in vivo* and *in vitro* datasets, a core gene list including 541 unique genes was generated. Based on the core gene list, further gene-disease associations, pathway and network analyses were performed. Data analyses based on multiple bioinformatics tools produced a large body of biologically meaningful information, and revealed a number of genes such as *SAMHD1, G6PD, GPD2* and *ENO1*, which have been reported to be related to immune response, blood biology, bone remodeling, and cancer respectively. As a unique resource, this study can serve as a reference map for future in-depth research on monocytes biology and monocyte-involved human diseases.

**Electronic supplementary material:**

The online version of this article (doi:10.1007/s13238-016-0342-x) contains supplementary material, which is available to authorized users.

## INTRODUCTION

The mission of the international Human Proteome Project (HPP) is to help elucidate biological and molecular function and advance diagnosis and treatment of diseases by systematically cataloging all proteins manufactured in the human body, and by generating a map of the protein-based molecular architecture of the human body.

Monocytes are a type of white blood cell derived from hemopoietic stem cells in bone marrow. They can usually be distinguished in stained smears by their large kidney shaped or notched nuclei. Monocytes play fundamental roles in human physiology; for example, as part of the human immune system, monocytes play a central role in regulating host inflammatory processes through chemotaxis, phagocytosis, and cytokine production (Jin et al., 2006). As precursors of bone-resorbing osteoclasts, monocytes are important for bone remodeling (Deng et al., 2011b; Zhou et al., 2015). Monocytes are also involved in muscle system processes (Grunberg et al., 2010), nervous system development (Verkman et al., 2011), regulation of tumor necrosis factor production (Kakazu et al., 2011). Despite the significance of monocytes in human physiology and pathology, the molecular bases underlying their diverse functions are poorly understood.

The human monocyte proteome has not yet been tackled by the Human Proteome Organization Initiative, and a global proteome database specifically for human monocytes has yet to be established. Quantitative proteomics, which is based on Liquid Chromatography (LC) and Mass Spectrometry (MS) technologies and can systematically identify and quantify proteins at a proteome-wide scale, has emerged as a novel and powerful methodology to characterize functions of various cell types and tissues in humans (Deng et al., 2011a). The purpose of this study is to present a comprehensive proteomic draft map for human monocytes. Dissecting the human monocyte proteome using modern quantitative proteomics methodologies will contribute to a comprehensive understanding of monocyte biology and the significance of monocytes in human health and diseases.

## Results

### Protein isoelectric point and molecular weight information

**Figure 1:**
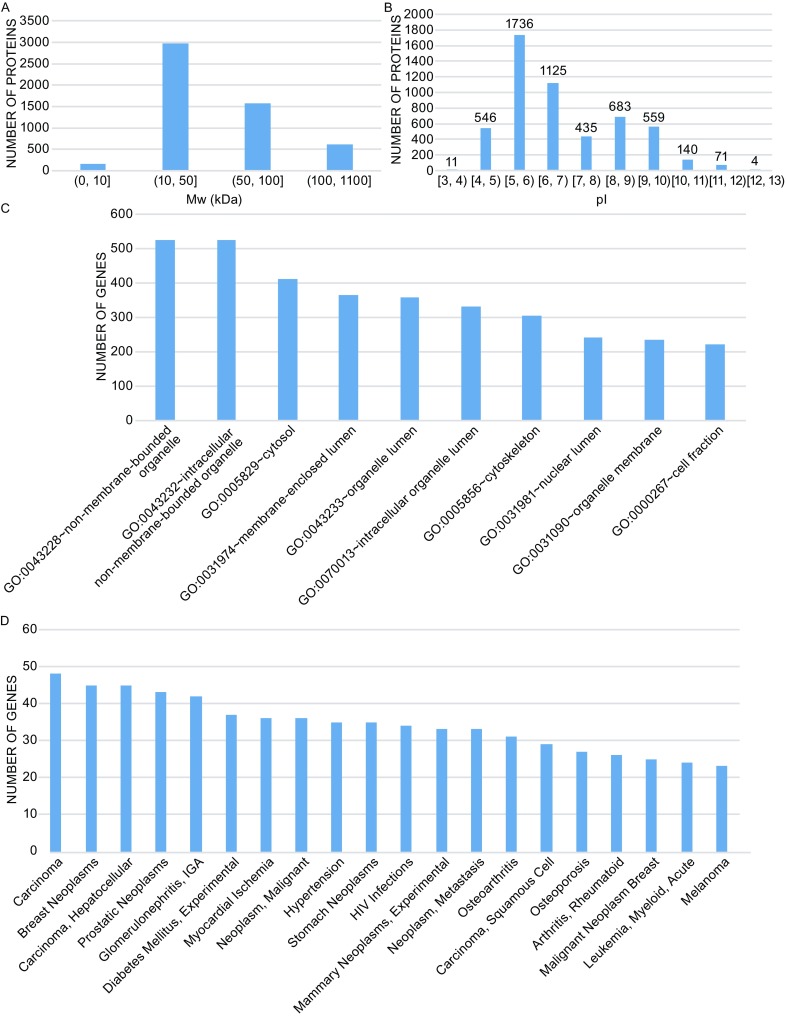
**Basic information and disease association of human monocyte proteins**. (A) Distribution of protein molecular weight. X-axis lists the specific Mw ranges. Y-axis shows the number of proteins distributed in each range. (B) Distribution of Protein Isoelectric Point. X axis lists the specific pI ranges. Y axis shows the number of proteins distributed in each range. (C) Top 10 terms in cellular component category. X axis shows the top 10 terms in the Cellular Component category. Y axis shows the number of genes enriched in each specific GO term. (D) 20 main diseases related to human monocytes. X axis shows the names of 20 diseases. Y axis shows the number of monocyte-expressed genes involved in each specific disease

Corresponding to 2060 genes out of the combined gene list, 5310 potential proteins went through the online tool for molecular weight (Mw) and isoelectric point (pI) calculation. The distributions of protein Mw and pI are shown in Fig. 1A and Fig. 1B respectively. It is well known that proteins are significant macromolecular substances in organisms. As expected, for most of the proteins, molecular weight values were larger than 10,000 Dalton. For protein pI distribution, as shown in Fig 1B), a large proportion of proteins were weakly acidic or weakly alkaline (6 < pI < 8). There were more acidic proteins than alkaline proteins (Table S3). This information may benefit future studies regarding human monocytes, especially for protein identification in electrophoresis experiments.

### Gene enrichment and functional annotation results

As displayed in Table S4, all the monocyte-expressed genes were annotated into a total of 13 categories and 2383 terms, derived from various databases such as GO, BIOCARTA, KEGG and others. All of the genes enriched in specific terms were listed. Meanwhile, for each term, a series of statistical values such as count and percentage of enriched genes, P value, fold enrichment score, FDR and Bonferroni value were calculated for the purpose of enrichment evaluation.

We looked into the most representative GO terms under several categories. In the cellular component (CC) category, based on the counts of enriched genes, Fig. 1C shows the top 10 terms illustrating the subcellular distribution of monocyte-expressed genes. As expected, most of the genes are enriched in cytosol, membrane, cytoskeleton and nuclear-related terms. In the molecular function (MF) category, based on the counts of enriched genes, Table 1 shows the top 20 terms involved in multiple functions such as nucleoside binding, ATP binding, RNA binding, calcium ion binding, and enzyme binding. In the biological process (BP) category, Table 2 highlights 20 terms which involved a variety of biological processes including immune response, defense response, protein localization and regulation of cell death. These 20 terms mainly represent the biological functions related to human monocytes.

**Table 1:** Top 20 terms in molecular function category

Terms	Number of genes	*P* value
GO:0000166~nucleotide binding	520	1.81E-44
GO:0017076~purine nucleotide binding	435	9.56E-34
GO:0032555~purine ribonucleotide binding	416	5.60E-32
GO:0032553~ribonucleotide binding	416	5.60E-32
GO:0001882~nucleoside binding	319	2.50E-14
GO:0001883~purine nucleoside binding	316	4.90E-14
GO:0030554~adenyl nucleotide binding	311	9.50E-14
GO:0032559~adenyl ribonucleotide binding	293	1.60E-12
GO:0005524~ATP binding	290	1.46E-12
GO:0005198~structural molecule activity	168	2.70E-19
GO:0003723~RNA binding	160	3.14E-11
GO:0005509~calcium ion binding	142	0.045
GO:0005525~GTP binding	136	6.68E-30
GO:0019001~guanyl nucleotide binding	136	1.43E-28
GO:0032561~guanyl ribonucleotide binding	136	1.43E-28
GO:0008092~cytoskeletal protein binding	136	3.09E-16
GO:0019899~enzyme binding	132	2.36E-13
GO:0042802~identical protein binding	131	4.65E-07
GO:0008233~peptidase activity	108	2.02E-04
GO:0070011~peptidase activity, acting on L-amino acid peptides	105	1.32E-04

Notes: Based on the number of enriched genes, the first column shows the names of the top 20 terms in the molecular function category. The second column shows the number of genes enriched in each specific term. The third column shows the p value of each term

**Table 2:** 20 main biological processes related to monocytes

Terms	Number of genes	*P* value
GO:0008104~protein localization	214	1.59E-20
GO:0045184~establishment of protein localization	200	6.49E-23
GO:0007242~intracellular signaling cascade	199	0.002737
GO:0015031~protein transport	198	1.23E-22
GO:0043933~macromolecular complex subunit organization	197	2.97E-26
GO:0006508~proteolysis	191	1.73E-06
GO:0065003~macromolecular complex assembly	187	1.14E-25
GO:0046907~intracellular transport	181	9.74E-24
GO:0006796~phosphate metabolic process	166	2.48E-04
GO:0006793~phosphorus metabolic process	166	2.48E-04
GO:0010941~regulation of cell death	162	3.28E-08
GO:0043067~regulation of programmed cell death	161	4.34E-08
GO:0042981~regulation of apoptosis	158	1.08E-07
GO:0006952~defense response	153	1.49E-15
GO:0016192~vesicle-mediated transport	151	9.85E-18
GO:0006955~immune response	151	1.10E-10
GO:0007049~cell cycle	151	4.01E-07
GO:0009611~response to wounding	150	6.20E-21
GO:0009057~macromolecule catabolic process	145	1.03E-05
GO:0010033~response to organic substance	143	2.71E-07

Notes: 20 terms which represent the main biological processes of monocytes are presented in the first column. The second column shows the number of genes enriched in each specific term. The third column shows the p value of each term

In order to gain a better understanding of monocyte-expressed proteins in human physiology and pathology, we intentionally looked into specific GO terms in the CC, MF and BP categories respectively to reveal genes which have been well recognized for their association with the immune system, blood, bone, cellular response, muscle, nervous system, and tumors, among others.

### Results of gene-disease association analysis

A total of 3444 gene-disease associations were obtained by matching the core gene list to the entire gene-disease association database. Among the associations, many diseases such as HIV infections, osteoporosis, arthritis, carcinoma and leukemia were highly associated with the physiology and pathology of human monocytes. Fig. 1D shows the number of genes involved in 20 main diseases. A single gene may be involved in different diseases; for example, *ANXA2* is involved in carcinoma, osteoporosis, and leukemia. Table S5 shows detailed information for all of these gene-disease associations, giving us a clear and comprehensive view of monocyte related diseases and providing a better understanding of the physiology and pathology of human monocytes.

### Results of pathway and network analysis

**Figure 2:**
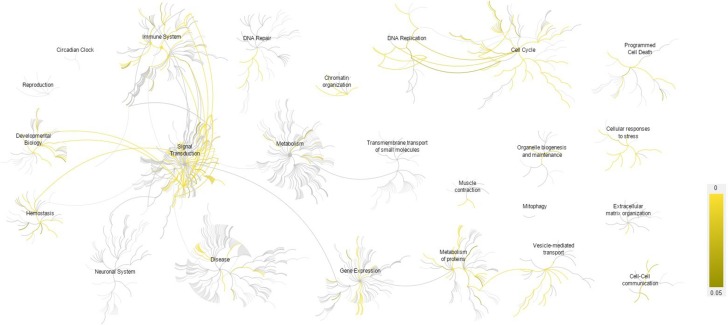
**Main groups and hierarchical relationship of Reactome pathways**. Reactome pathways are classified into different groups based on the similarity of their biological functions. The bottom color gradation provides the *P* value comparison for significant pathways. The dendritic structure represents the hierarchical relation of pathways

**Figure 3:**
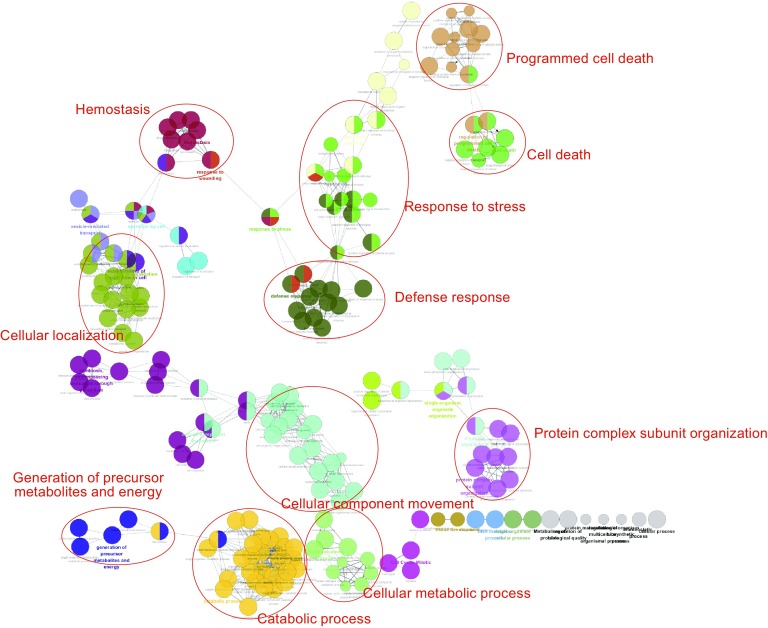
**Network reconstruction of functional terms**. Each dot represents one functional term. The same color dots represent terms which have similar biological functions. Accordingly, all terms were classified into different functional modules including “defense response,” “response to stress,” “hemostasis” and “cellular localization”

The core gene list, including 541 unique genes, was put through the Reactome online tool for pathway analysis. Accordingly, a total of 972 pathways as well as a series of statistical values including “Entities pValue” and “Entities FDR” were generated (Table S6). Based on physiological similarity and hierarchy, pathways were classified into several groups including immune system, signal transduction, metabolism, disease, cell cycle and hemostasis (Fig. 2). Similarly, in network analysis based on Cytoscape plugins, all provided genes were first enriched in specific terms, then all the terms were classified into 30 functional modules (S7 Table). As shown in Figs. 3 and S1, the genes shown to be involved in biological processes including “defense response,” “cellular localization,” “hemostasis,” “response to stress,” “cellular component movement,” and “catabolic process” make up a large proportion of all provided monocyte-expressed genes.

## DISCUSSION

Human monocytes play fundamental roles in human physiology, including immune system processes, blood coagulation and circulation, bone remodeling and resorption, muscle system processes and nervous system development (see details in Table S4). Dissecting global monocyte proteomics may offer a fresh perspective into understanding monocyte-related biological process and pathology in humans. In this study, we developed the first proteome knowledge base specifically for human monocytes. Such a knowledge base, integrating *in vivo* monocyte proteome datasets from human PBM samples and *in vitro* proteome datasets from cultured monocytes, may represent a comprehensive knowledge base for monocyte-expressed proteins in human population. We cataloged a total of 2237 unique monocyte-expressed genes, and conveyed serial information for these genes and their corresponding proteins including protein biochemical features (Mw and pI), protein functions (cellular component, molecular function, and biological processes), gene-disease association and pathway/network information.

Monocytes can act as precursors of macrophages and dendritic cells in the human immune system. Macrophages and dendritic cells have key roles in antigenic infections, linking innate antigen detection to antigen adaptive immune responses (Hrecka et al., 2011). In our study, GO analysis results indicated that 151 monocyte-expressed genes are enriched in the “immune response” term. At the same time, Reactome pathway analysis showed that 134 genes are enriched in the pathway “immune system.” More importantly, in the gene-disease association study, a large number of genes are involved in immunological diseases such as HIV infections, arthritis and other autoimmune diseases. Among these genes, *SAMHD1*, *NCF1*, *NCF2*, and *CD14* have been demonstrated to be involved in many kinds of human immune related physiology pathways or diseases. *SAMHD1* has a protective role in preventing self-activation of innate immunity by cell-intrinsic components (Hrecka et al., 2011). Evidence indicates that *SAMHD1* directly participates in HIV-1 infection macrophage mediation (Hrecka et al., 2011). Meanwhile, as a restriction factor, *SAMHD1* has been proposed to inhibit HIV-1 by blocking viruses at different steps of their life cycle (Merindol and Berthoux, 2015). Our gene-disease association study supports the theory that *NCF1* and *NCF2* are associated with some immunological diseases including arthritis and granulomatous disease. Previous evidence has demonstrated that mutations in *NCF1* and *NCF2* may cause granulomatous disease, and the copy number variation of *NCF1* is associated with rheumatoid arthritis (de Boer et al., 2002; Koker, 2010; Olsson et al., 2012). Previous studies have also reported that *CD14* deficiency increased clinical symptoms in active experimental autoimmune encephalomyelitis (Walter et al., 2006). Furthermore, as a marker of monocyte activation, soluble *CD14* is associated with morbidity and mortality in HIV disease (Shive et al., 2015). Consistently, our gene-disease association study denoted that *CD14* is involved in many immunological diseases such as HIV infections, inflammation and some autoimmune diseases. These examples demonstrate that monocytes are a fundamental part of the human immune system and play an irreplaceable role in immune-associated biological processes in humans.

Monocytes are also involved in different kinds of blood-associated biological processes including blood coagulation, blood circulation, and leukocyte migration. Accordingly, as listed in S5 Table, monocyte-expressed genes are involved in several blood-associated diseases such as leukemia, anemia, blood coagulation and blood platelet disorders. Herein, we highlighted several genes such as *G6PD*, *ITGB2* and *ANXA5* which were sorted out from blood related terms including “hemostasis,” “blood coagulation,” “leukocyte migration” and “hereditary hemolytic anemia.” *G6PD* deficiency is the most common metabolic disorder of red blood cells, affecting about 35 million people worldwide (Nabavizadeh and Anushiravani, 2007). An atomic force microscopy study suggested that *G6PD* plays a significant role in hemolytic anemia by affecting the membrane structure of red blood cells (Tang et al., 2015). The severity of clinical manifestations of myeloid leukemia is directly related to the degree of *CD18* (*ITGB2*) deficiency (Vasconcelos Dde et al., 2014). As a biochemical marker of atherosclerosis, the concentration of *ANXA5* serves as a standard for clinical diagnosis of cardiovascular complications in kidney disease (Emanuel et al., 2013). These examples highlight the close relationship between monocyte-expressed genes/proteins and blood physiology in humans.

Monocytes can serve as a precursor of bone-resorbing osteoclasts. Monocytes may access bone resorption surfaces to differentiate into osteoclasts to resorb bone, which is important for bone remodeling and fracture repair (Deng et al., 2011a). Many proteins related to bone formation or resorption mechanism thus involved in osteoporosis were identified. Herein, four genes, *GPD2*, *ANXA2*, *TPM4* and *GSN*, have been selected for subsequent discussion according to functional annotation and gene-disease association analysis in our study. These four genes are sorted out from the term “calcium ion binding” and are all involved in osteoporosis in gene-disease association analysis. Previous studies from our group have supported the contribution of *GPD2*, *TPM4* and *GSN* to osteoporosis risk (Deng et al., 2008; Deng et al., 2014). Several previous studies suggested that *ANXA2* can influence bone formation in a variety of ways including regulation of osteoprogenitor proliferation, differentiation, and responsiveness to cytokines (Deng et al., 2011a; Pandey et al., 2012; Genetos et al., 2014). These examples suggest that monocytes are functionally relevant to bone. Extensive studies on monocyte-expressed proteins may provide novel insights into the interaction between monocytes and bone.

Monocytes are involved in a variety of human physiological functions and associated with many complex human diseases (Dichamp et al., 2007; Chimma et al., 2009; Kraft-Terry et al., 2010). Five diseases closely related to the physiology and pathology of monocytes are presented in Table 3, along with a list of genes involved in 2 or more diseases. In-depth studies of monocyte-expressed proteins will contribute to a better understanding of the molecular pathophysiological mechanisms underlying these diseases. The *in vivo* protein expression data based on LC-MS may serve as a reference map and use for validation study for functional research on individual monocyte proteins. Basic information including molecular weight and isoelectric point is important for electrophoretic experiment focus on specific monocyte protein. Results from multiple bioinformatics analysis allow us to better understand the interaction between monocyte proteins, which may help other researchers to develop new ideas on physiology and etiology studies regarding human monocyte. In summary, our study provided a reference map for future in-depth research on monocytes and monocyte-involved human diseases, which may facilitate the understanding of monocyte biology and monocyte-related human health and diseases for the research community.

**Table 3:** Five representative diseases and the involved genes related to monocytes

	Arthritis	Carcinoma	HIV infections	Leukemia	Osteoporosis
HLA-A	1	1	1	1	
HLA-B	1	1	1	1	
HLA-C	1	1	1	1	
CAT	1		1		1
ENO1	1	1			1
GSTP1	1	1		1	
SOD2	1	1			1
ACTG1		1			1
ANXA1	1	1			
ANXA2				1	1
ANXA4		1		1	
ATIC	1				1
CD14	1		1		
CDC42		1	1		
HLA-DRB5	1		1		
HSPD1	1		1		
IDH2				1	1
ITGB2	1			1	
LGALS3	1	1			
MPO	1			1	
MVP		1		1	
NME1		1		1	
PLEK	1				1
PRTN3	1			1	
PSMA4		1	1		
PSMA5			1		1
PTPRC	1		1		
RAN		1	1		
S100A8	1			1	
SERPINA1	1		1		
TAP1	1	1			
VIM	1	1			

Notes: In this table, 5 diseases closely related to human monocytes are selected and shown in the first row. Among these 5 diseases, genes involved in 2 or more diseases are listed in the first column. The number “1” means that the corresponding gene is involved in the specified disease

## MATERIAL AND METHODS

### *In vivo* data acquisition

#### Human subjects

The study was approved by the Institutional Review Boards of University of Missouri Kansas City and Tulane University. A total of 76 unrelated female Caucasians (Age: 57.8 ± 9.0) were involved in this study. Signed informed consent documents were obtained from all study participants before they were enrolled.

#### Peripheral blood monocyte (PBM) isolation

For *in vivo* data, monocytes were extracted from human peripheral blood. About 60ml of peripheral blood was collected from each subject by certificated phlebotomists. Ethylenediaminetetraacetic acid (EDTA) was used as an anti-coagulant. The fresh blood samples were processed instantly for PBM isolation by experienced technicians. First, peripheral blood mononuclear cells (PBMC) were isolated from whole blood using density gradient centrifugation with Histopaque-1077 (Sigma, Catalog No. H1077-1). Then, PBM were isolated from PBMC using a monocyte negative isolation kit (Dynal Biotech Inc.) following the manufacturer’s recommendation. The kit contains a highly optimized antibody mix, blocking reagent, and Depletion Dynabeads to deplete T cells, B cells, and natural killer cells from PBMC, leaving monocytes untouched and free of surface-bound antibodies and beads. Per our experience, the purity of PBM isolated using this method was 86% ± 3% (Liu et al., 2005).

#### Protein extraction and sample preparation

PBM total proteins were extracted using a complete proteome extraction mammalian kit (Calbiochem Catalog No. 539779). Protein concentration was measured using the Bradford method. Up to 20 μg total protein was precipitated using a protein precipitation kit (Calbiochem Catalog No. 539180). Protein pellets were dissolved in 50 μl. 50 mmol/L ammonium bicarbonate with 0.1% RapiGest (Waters), reduced by 5.0 mmol/L dithiothreitol, alkylated by 15 mmol/L indole acetic acid, then digested by trypsin (Sigma, Catalog No.T6567). Protein digests were concentrated to approximately 20 μL, of which 15 μL was aspirated and brought back to 20 μL, with 0.5% formic acid (FA) and 100 fmol yeast ADH1 (alcohol dehydrogenase I) digestion standard (Waters, Catalog No. 186002328).

#### PBM proteome profiling through LC-nano-ESI-MS/MS^E^ analyses

PBM proteomes were profiled using the method of LC-nano-ESI-MS^E^, through nanoAcquity Ultra Performance Liquid Chromatography coupled with Synapt High Definition Mass Spectrometry (HDMS) (Waters Corporation). Proteome data acquisition was controlled by MassLynx4.1 software (Waters). Briefly, the protein digests (~ 500 ng) were injected into a BEH C18 75 μm × 150 mm analytical column (Waters), and separated by solvent A (water with 0.1% FA) and solvent B (acetonitrile with 0.1% FA) at a flow rate of 0.3 μL/min using a 2-h gradient. The eluate was analyzed by HDMS under positive ion V-mode. The following parameters were set for data acquisition: collision energy: 5 volts for MS and ramp 15–40 volts for MS^E^; scan time: 0.6 second per scan. The HDMS machine was calibrated daily to ensure high accuracy (2.0 ppm for lock mass of m/z 785.8426).

For each PBM proteome digest sample, triplicate LC-nano-ESI-MS^E^ datasets were acquired, The MS^E^ data were then processed with ProteinLynx Global Server v2.3 (Waters) using default parameters. Based on the alternating low- and elevated-energy nature of MS^E^ data, properties of each ion (mass-to-charge ratio, retention time, intensity, etc.) were determined, and a list of all precursor and product ions was produced. Specifically, the ion’s intensity was derived from the areas of both the chromatographic and mass spectrometric peaks. The precursor ion intensity threshold was set to above 1000 counts. The human protein database International Protein Index v3.83 (153,078 protein entries) was searched by using the following parameters: enzyme specificity: trypsin; number of missed cleavages permitted: 1; fixed modification: Carbamidomethyl C; variable modifications: Acetyl N-TERM, Deamidation N, Deamidation Q, and Oxidation M; mass tolerance for precursor ions: 15 ppm; mass tolerance for product ions: 30 ppm; minimum peptide matches per protein: 1; minimum fragment ion matches per protein: 7; minimum fragment ion matches per peptide: 3; false positive rate: limited to 4% per randomized database searching. For each PBM sample, only proteins identified at least twice in the triplicate LC-nano-ESI-MSE analyses were reported as truly present. Total ion counts of the three most intensely matched peptides were used to quantify each protein. With the standard ADH1 as references, protein quantification level was exported in femtomol and nanogram. Mean values from triplicate analyses were used to represent protein expression levels in each PBM sample.

### *In vitro* dataset

The *in vitro* dataset was downloaded from the Human Proteome Map (HPM) project (http://www.humanproteomemap.org/), which was published in *Nature* in 2014 by Kim et al (Kim et al., 2014) with great effort and collaboration from 20 research institutions. The project was based on LC-MS/MS and utilized high resolution and high accuracy Fourier transform mass spectrometry (LTQ-Orbitrap Elite and LTQ-Orbitrap Velos). The authors obtained numerous comprehensive datasets from multiple organs, tissues and cell types including 17 adult tissues, 6 primary hematopoietic cells and 7 fetal tissues.

For the specific purpose of our study, we used only the dataset obtained from monocytes. The monocytes used in their study were harvested from cell culture; hence, they provided *in vitro* results and contributed a significant supplemental dataset for our study. The top 2000 monocyte-expressed genes from the *in vitro* results were selected for the integrated proteomics in our study.

### Protein isoelectric point and molecular weight calculation

By integrating both *in vivo* and *in vitro* data, a combined gene list including 2237 unique genes was generated **(Table S1**). Based on the combined gene list, we first converted the gene IDs to protein IDs (UniProt Accession Number) to meet the requirement of the online tool, then calculated the protein isoelectric point (pI) and molecular weight (Mw) for all the corresponding proteins using the Compute pI/Mw tool provided by Expert Protein Analysis System (ExPASy, http://web.expasy.org/compute_pi/). It should be noted that one gene may correspond to multiple proteins due to the variety in translation and protein modification.

### Functional annotation and gene enrichment analysis

The DAVID (Database for Annotation, Visualization and Integrated Discovery) Knowledgebase has agglomerated tens of millions of gene/protein identifiers from a variety of public genomic resources. These identifiers improve cross-referencing capability, particularly across NCBI and UniProt systems. More than 40 publicly available functional annotation sources have been comprehensively integrated and centralized by the DAVID gene clusters (Sherman et al., 2007; Huang et al., 2009). As mentioned above, the combined gene list including 2237 unique genes was imported into this online tool for functional annotation and enrichment analysis. Comprehensive functional information for all genes enriched in specific terms has been fully exposed. The results were classified as “functional annotation clustering,” “functional annotation chart” and “functional annotation table.”

### Gene-disease association analysis

Although the *in vivo* and *in vitro* datasets were generated from different experimental materials as well as different instruments, they both stem from the same cell type: monocyte. By extracting the overlapped genes between *in vivo* and *in vitro* data, a core gene list including 541 unique genes was generated (Table S2). With high repeatability and reliability between the independent *in vivo* and *in vitro* experiments, the overlapped genes are most likely to reflect the biological functions related to human monocyte exactly and objectively. Therefore, we performed gene-disease association analysis on the basis of the core gene list. First, we downloaded the whole gene-disease association dataset for the DisGeNET database (http://www.disgenet.org/web/DisGeNET/menu/home) which contains about 50 thousand associations between different genes and diseases. The associations are all supported by experimental evidence or previous publications. Secondly, we mapped our core gene list to the entire knowledge base, so that all of the gene-disease associations corresponding to the provided genes could be extracted; one gene may be involved in several diseases, and one disease may involve numerous genes.

### Pathway and network analysis

Network analysis can characterize the functional correlation and interaction topology between different proteins. Pathways/modules generated from network analysis may reflect biological processes more comprehensively and objectively than from a single protein or gene (Swa et al., 2014). Reactome (http://www.reactome.org/) is a free, open-source, curated and peer-reviewed pathway database which provides multiple bioinformatics tools for the visualization, interpretation and analysis of pathway knowledge. Because of these advantages, we imported the core gene list into Reactome online tools for pathway analysis. The core gene list was also imported into the plugin ClueGO in conjunction with CluePedia for network analysis and visualization (Bindea et al., 2009; Bindea et al., 2013). The topological information including interactions between genes and pathways are shown in tables and charts.

### Data source

We uploaded all the result files regarding the data sets as supplemental files (Tables S1–S8 and Fig. S1). They include *in vivo* protein expression data (Table S8), the combined and overlapped gene list corresponding to both *in vivo* and *in vitro* proteomics data (Tables S1 and S2), basic characteristics including molecular weight and isoelectric point of human monocyte proteins (Table S3), results from multiple bioinformatics analyses (Tables S4–S7 and Fig. S1). Readers can review, download, and use the data from the online version paper directly.

## Electronic supplementary material

Below is the link to the electronic supplementary material.

Supplementary material 1 (XLSX 36 kb)

Supplementary material 2 (XLSX 14 kb)

Supplementary material 3 (XLSX 201 kb)

Supplementary material 4 (XLSX 378 kb)

Supplementary material 5 (XLSX 66 kb)

Supplementary material 6 (XLSX 188 kb)

Supplementary material 7 (XLSX 16 kb)

Supplementary material 8 (XLSX 911 kb)

Supplementary material 9 (PDF 212 kb)
