# Supplementary material for: Mass spectrometry based proteomics profiling of human monocytes
Source: Protein Cell. 2016 Nov 22;8(2):123–33. doi: 10.1007/s13238-016-0342-x (PMC5291777; doi:10.1007/s13238-016-0342-x)
Supplement: Supplementary file 9 — Supplementary material 9 (PDF 212 kb) [file 13238_2016_342_MOESM9_ESM.pdf]

Supplementary data

Table S1. Combined Gene List

Table S2. Core Gene List

Table S3. PI and MW Information

Table S4. Gene Enrichment and Functional Annotation

Table S5. Gene-Disease Associations

Table S6. Reactome Pathways

Table S7. Network Results

Table S8. In vivo protein expression data

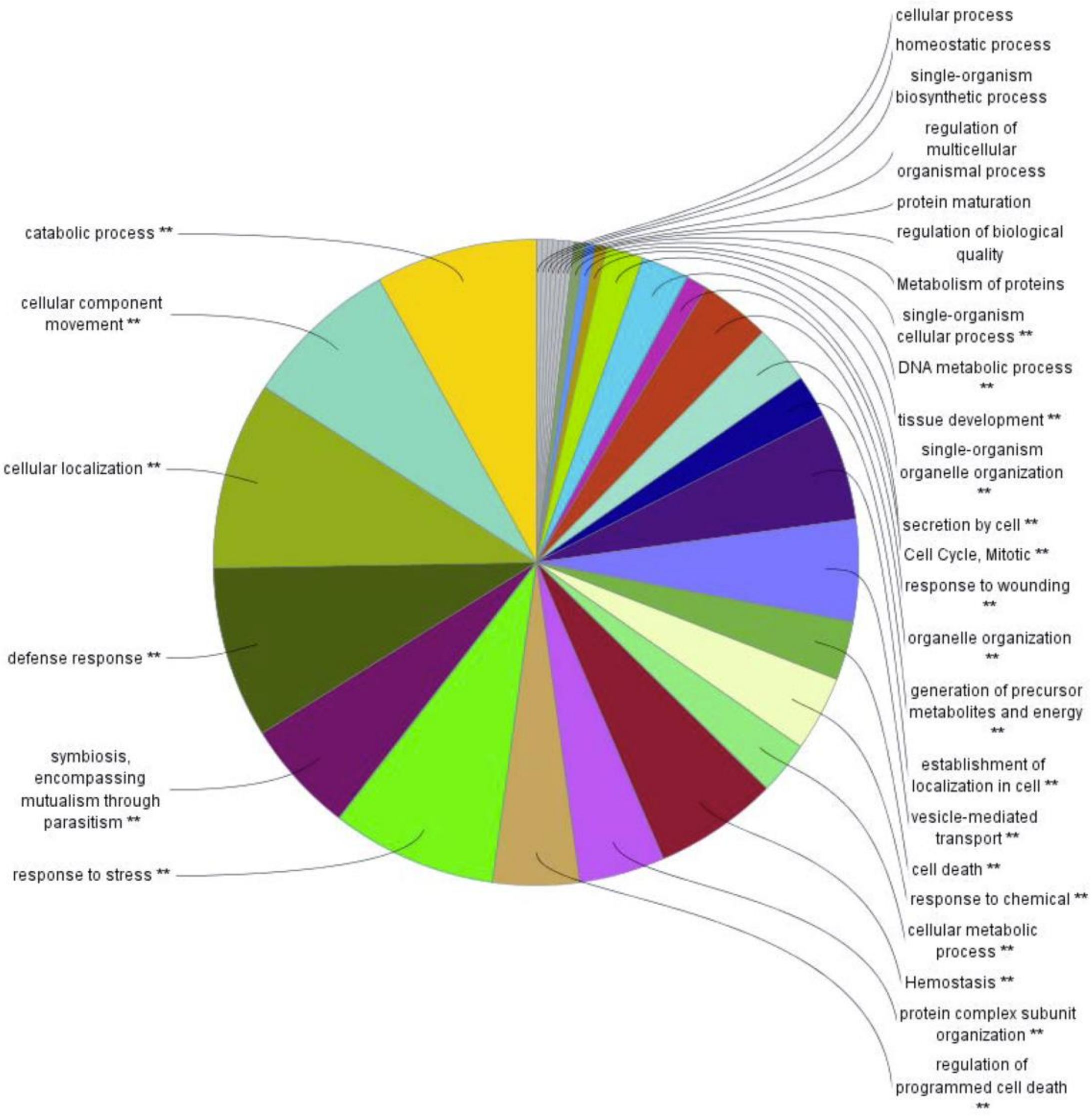

Figure S1. Overview of specific functional modules.
